# The genome sequence of the Downland Villa bee-fly, *Villa cingulata *(Meigen, 1804)

**DOI:** 10.12688/wellcomeopenres.20123.1

**Published:** 2023-11-14

**Authors:** Susan C. Taylor, Liam M. Crowley, Sally Luker, Martin Harvey, Natural History Museum Genome Acquisition Lab, University of Oxford and Wytham Woods Genome Acquisition Lab, Darwin Tree of Life Barcoding collective, Wellcome Sanger Institute Tree of Life programme, Wellcome Sanger Institute Scientific Operations: DNA Pipelines collective, Tree of Life Core Informatics collective, Darwin Tree of Life Consortium

**Affiliations:** 1 Dipterists Forum, Northchurch, England, UK; 2 University of Oxford, Oxford, England, UK; 3 University of Exeter, Penryn, England, UK; 4 UK Centre for Ecology & Hydrology, Wallingford, England, UK

**Keywords:** Villa cingulata, Downland Villa Bee-fly, genome sequence, chromosomal, Diptera

## Abstract

We present a genome assembly from an individual male *Villa cingulata* (the Downland Villa bee-fly; Arthropoda; Insecta; Diptera; Bombyliidae). The genome sequence is 412.6 megabases in span. Most of the assembly is scaffolded into 10 chromosomal pseudomolecules, including the X and Y sex chromosomes. The mitochondrial genome has also been assembled and is 22.43 kilobases in length.

## Species taxonomy

Eukaryota; Metazoa; Eumetazoa; Bilateria; Protostomia; Ecdysozoa; Panarthropoda; Arthropoda; Mandibulata; Pancrustacea; Hexapoda; Insecta; Dicondylia; Pterygota; Neoptera; Endopterygota; Diptera; Brachycera; Muscomorpha; Asiloidea; Bombyliidae; Anthracinae; Villini; *Villa*; *Villa cingulata* (Meigen, 1804) (NCBI:txid2753613).

## Background

The genus *Villa* Lioy 1864 comprises more than 270 described species globally ( Evenhuis, 1999; Evenhuis & Greathead, 2015; GBIF Secretariat, 2022; Hull, 1973) of short-tongued bee-flies (Diptera: Bombyliidae). *Villa* spp. are considered parasites of Lepidopteran species, notably of Noctuid moths (Lepidoptera, Noctuidae). As with other bee-flies, while in flight, females flick single, dust-covered eggs in the direction of their hosts, which then develop inside the larvae/pupae ( Falck, 2009; Stubbs & Drake, 2014). *Villa cingulata* (Meigen, 1804) is commonly known as the Downland Villa; it is one of three *Villa* species known from the UK, the other two being *V. modesta* (Meigen, 1820) and *V. venusta* (Meigen, 1820). *V. venusta* is presumed extinct in the UK ( Drake, 2017).

A photographic identification guide to the UK *Villa* spp. has been compiled for the Soldierflies and Allies Recording Scheme ( Harvey, 2022), and a detailed description and key is provided in Stubbs and Drake (2014). All three UK species are medium-to-large flies with a characteristic overall appearance of a fairly broad, flat abdomen, clear wings with dark bases, and varying degrees and arrangements of golden hairs and dark scales ( Harvey, 2022; Stubbs & Drake, 2014). Differences in abdominal banding and extent of darkened wings are particularly useful in separating the species, with all three exhibiting varying degrees of sexual dimorphism.

While the three UK Villa species are very similar in appearance, clear differences in the associated habitats and current distributions of each are currently observed, with *V. cingulata* showing a preference for inland grasslands. Until quite recently *V. cingulata* was very predominantly an insect of calcareous meadows and chalk downland with short turf and/or minimal vegetation cover ( Harvey, 2022; Stubbs & Drake, 2014). However, since 2013 it has also been found in neutral hay meadow habitats (e.g. Chandler, 2015) and in the last few years has also colonised some acid grasslands, e.g. in the New Forest ( Soldierflies and Allies Recording Scheme, 2023). Some of these recent sites have tall grassland swards, but paths and patches of bare ground are usually present as well.

Historically recorded, albeit in very low numbers, from several southern UK counties, where it was described as occurring on shrubby, chalky slopes with tall vegetation ( Drake, 2017; Stubbs & Drake, 2014; Verrall, 1909). A period of about 60 years with no records of *V. cingulata* led to concerns that the species might be extinct in the UK; however, in 2000, the species was rediscovered in the Cotswolds, with additional records from nearby areas subsequently reported ( Stubbs & Drake (2014) and references therein). The threat status for *V. cingulata* was assessed as “Least Concern” by Drake (2017), and at that time it was given a rarity status of Nationally Rare. However, that assessment was based largely on data prior to 2016, and since then the number of records per year has greatly increased, as has the number of occupied ten-kilometre squares, and the species would no longer qualify as Nationally Rare based on current data. Since 2000 it has been recorded from 21 vice-counties in southern England and south-east Wales ( Soldierflies and Allies Recording Scheme, 2023).

Adults of *V. cingulata* are found in summer, from June to August ( Harvey, 2022; Stubbs & Drake, 2014). Adult flies can be observed visiting umbellifers (Fam. Apiaceae), such as Wild Parsnip and Hogweed, while females will often rest on warm ground, gathering dust on their abdomens, which subsequently adheres to their eggs to enable effective dispersal via flicking ( Falck, 2009). The host of *V. cingulata* in the UK remains unknown (e.g. Stubbs & Drake, 2014). Males have been observed to be territorial/defensive to conspecific males ( Falck, 2009).

The specimen was collected in July 2021. On the continent *V. cingulata* is found throughout Europe and the Middle East ( Evenhuis & Greathead, 2015).

The genome of *V. cingulata* was sequenced as part of the Darwin Tree of Life Project, a collaborative effort to sequence all named eukaryotic species in the Atlantic Archipelago of Britain and Ireland. Here we present a chromosomally complete genome sequence for *Villa cingulata*, based on one male specimen from Holtspur Bottom Nature Reserve, UK.

## Genome sequence report

**Figure 1.**
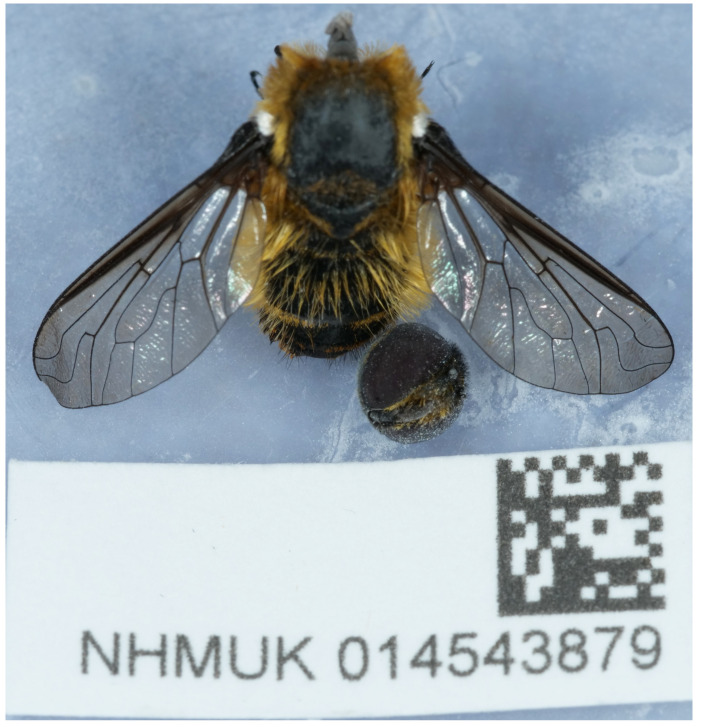
Photograph of the *Villa cingulata* (idVilCing2) specimen used for genome sequencing.

The genome was sequenced from one male *Villa cingulata* ( Figure 1) collected from Holtspur Bottom Nature reserve, England, UK (51.61, –0.67). A total of 54-fold coverage in Pacific Biosciences single-molecule HiFi long reads was generated. Primary assembly contigs were scaffolded with chromosome conformation Hi-C data. Manual assembly curation removed contaminating scaffolds and corrected 5 missing joins or mis-joins and removed one haplotypic duplication, reducing the assembly length by 47.54% and the scaffold number by 99.35%, and increasing the scaffold N50 by 221.12%.

**Figure 2.**
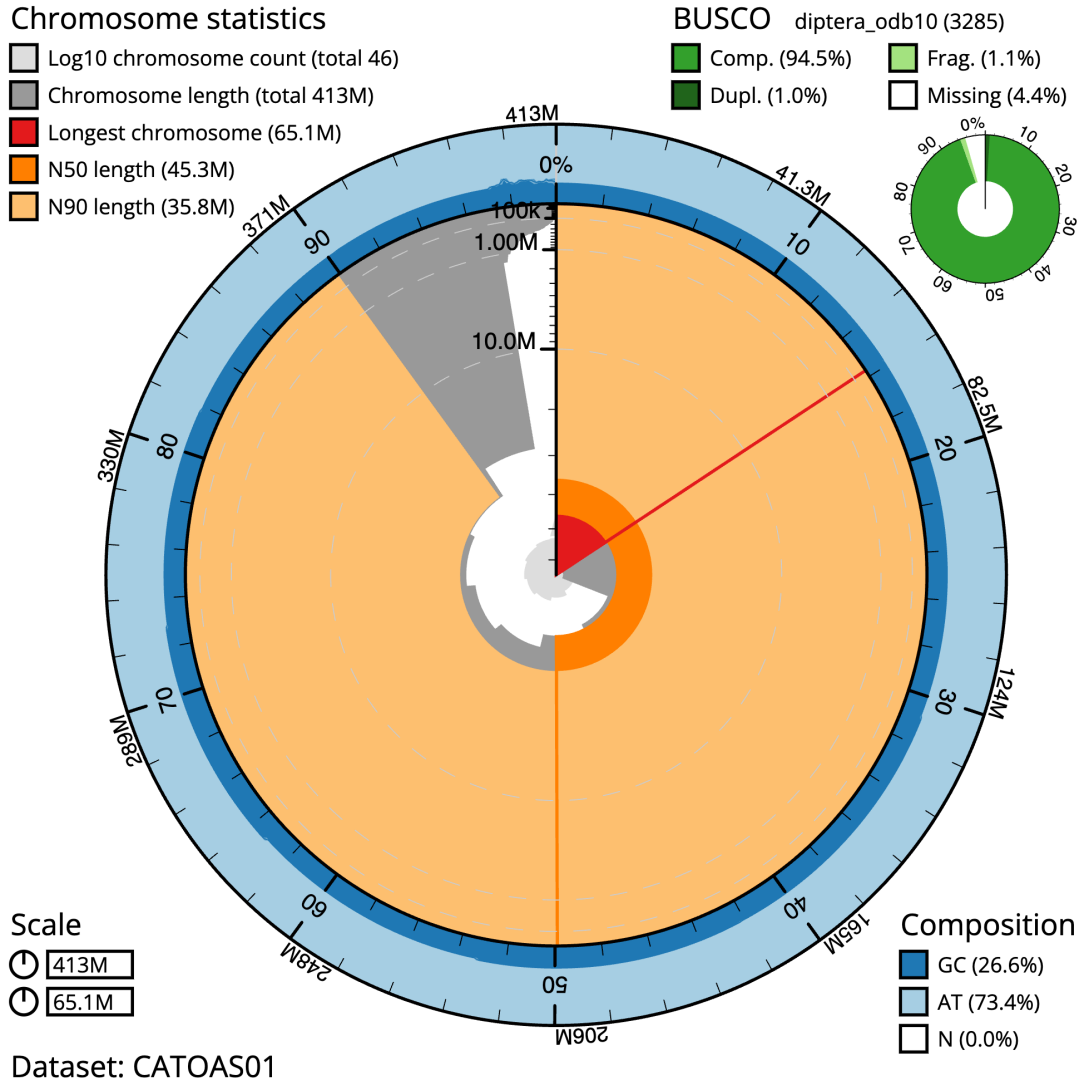
Genome assembly of *Villa cingulata*, idVilCing2.1: metrics. The BlobToolKit Snailplot shows N50 metrics and BUSCO gene completeness. The main plot is divided into 1,000 size-ordered bins around the circumference with each bin representing 0.1% of the 412,610,433 bp assembly. The distribution of scaffold lengths is shown in dark grey with the plot radius scaled to the longest scaffold present in the assembly (65,149,795 bp, shown in red). Orange and pale-orange arcs show the N50 and N90 scaffold lengths (45,252,737 and 35,763,846 bp), respectively. The pale grey spiral shows the cumulative scaffold count on a log scale with white scale lines showing successive orders of magnitude. The blue and pale-blue area around the outside of the plot shows the distribution of GC, AT and N percentages in the same bins as the inner plot. A summary of complete, fragmented, duplicated and missing BUSCO genes in the diptera_odb10 set is shown in the top right. An interactive version of this figure is available at https://blobtoolkit.genomehubs.org/view/idVilCing2.1/dataset/CATOAS01/snail.

**Figure 3.**
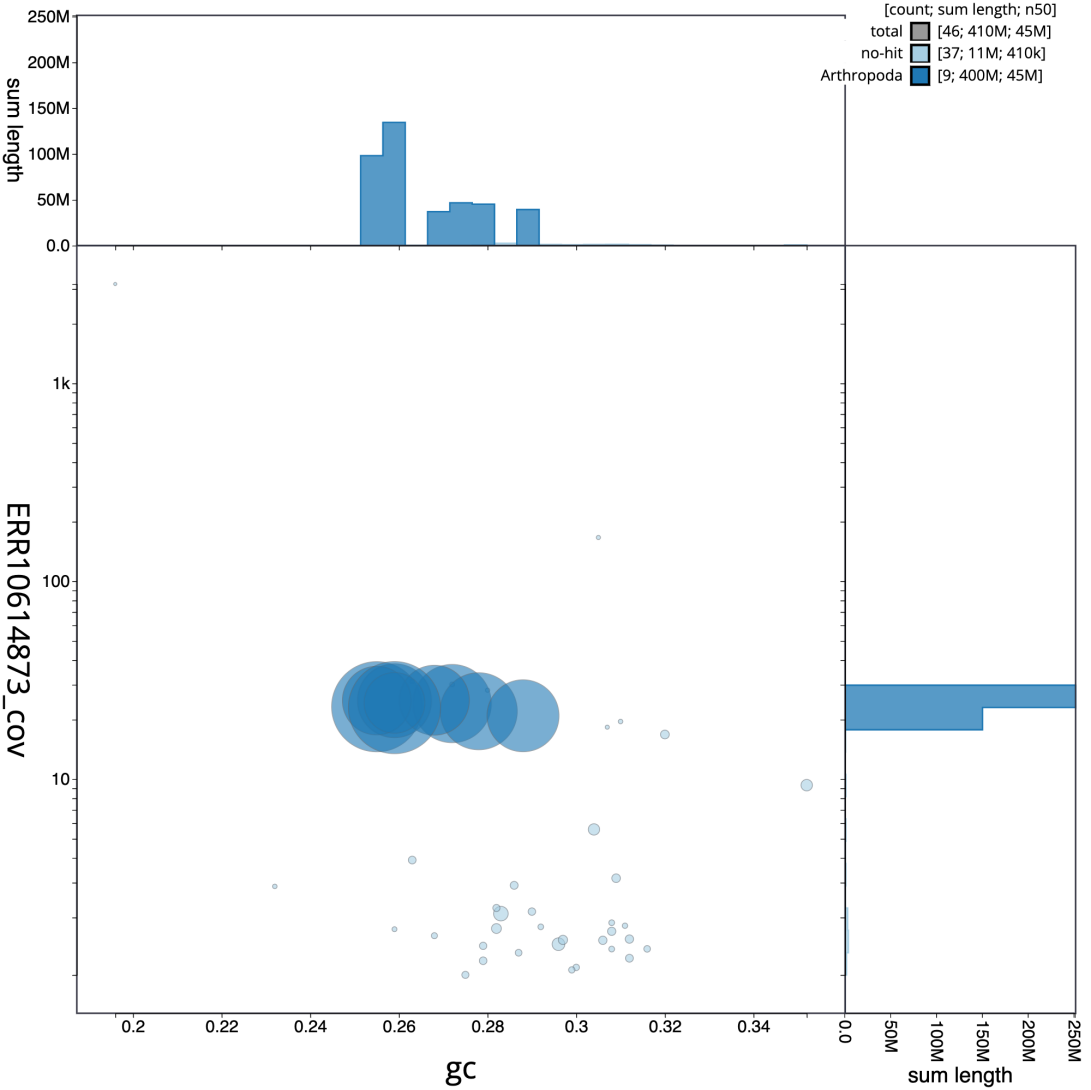
Genome assembly of *Villa cingulata*, idVilCing2.1: BlobToolKit GC-coverage plot. Scaffolds are coloured by phylum. Circles are sized in proportion to scaffold length. Histograms show the distribution of scaffold length sum along each axis. An interactive version of this figure is available at https://blobtoolkit.genomehubs.org/view/idVilCing2.1/dataset/CATOAS01/blob.

**Figure 4.**
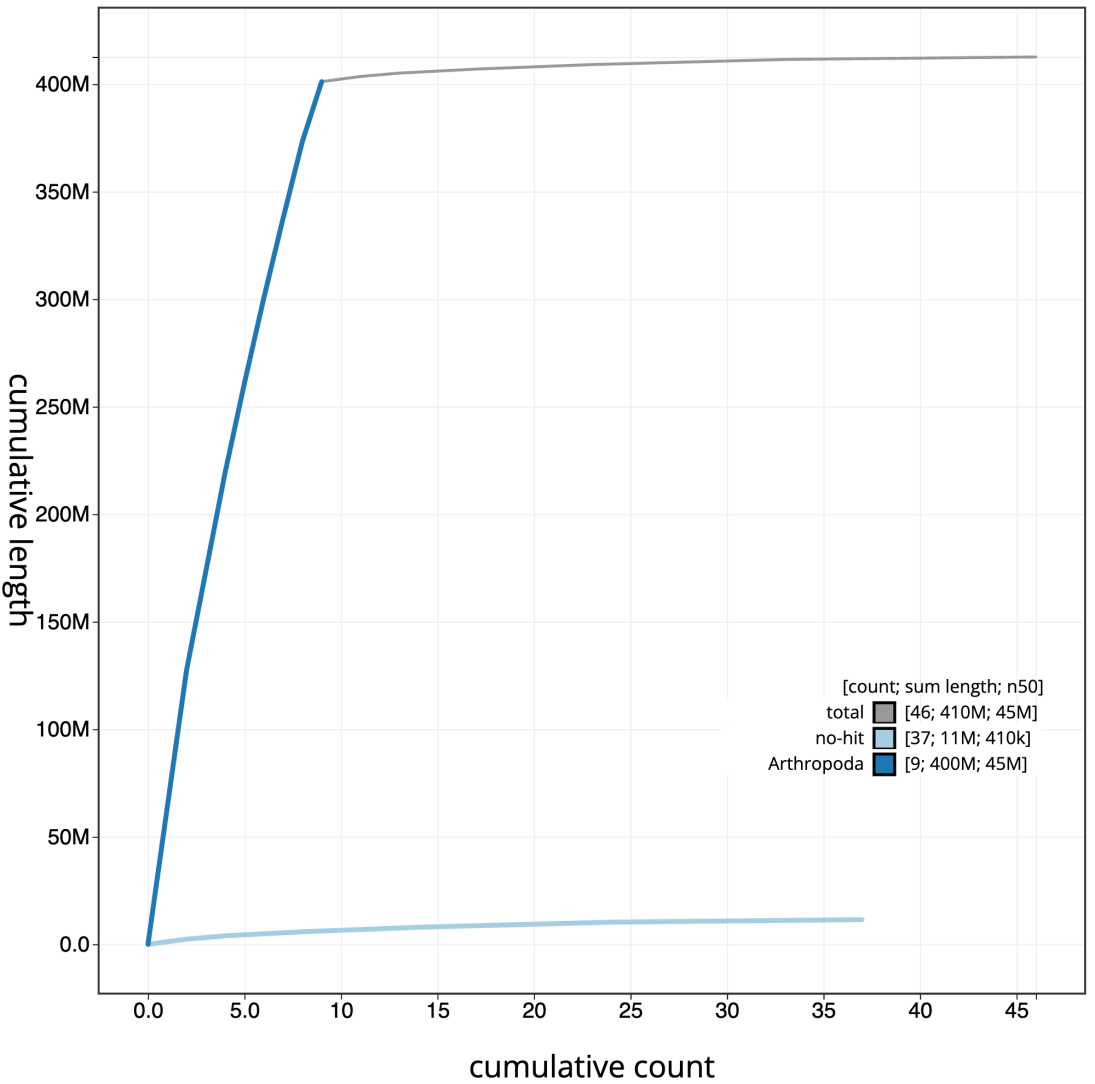
Genome assembly of *Villa cingulata*, idVilCing2.1: BlobToolKit cumulative sequence plot. The grey line shows cumulative length for all scaffolds. Coloured lines show cumulative lengths of scaffolds assigned to each phylum using the buscogenes taxrule. An interactive version of this figure is available at https://blobtoolkit.genomehubs.org/view/idVilCing2.1/dataset/CATOAS01/cumulative.

**Figure 5.**
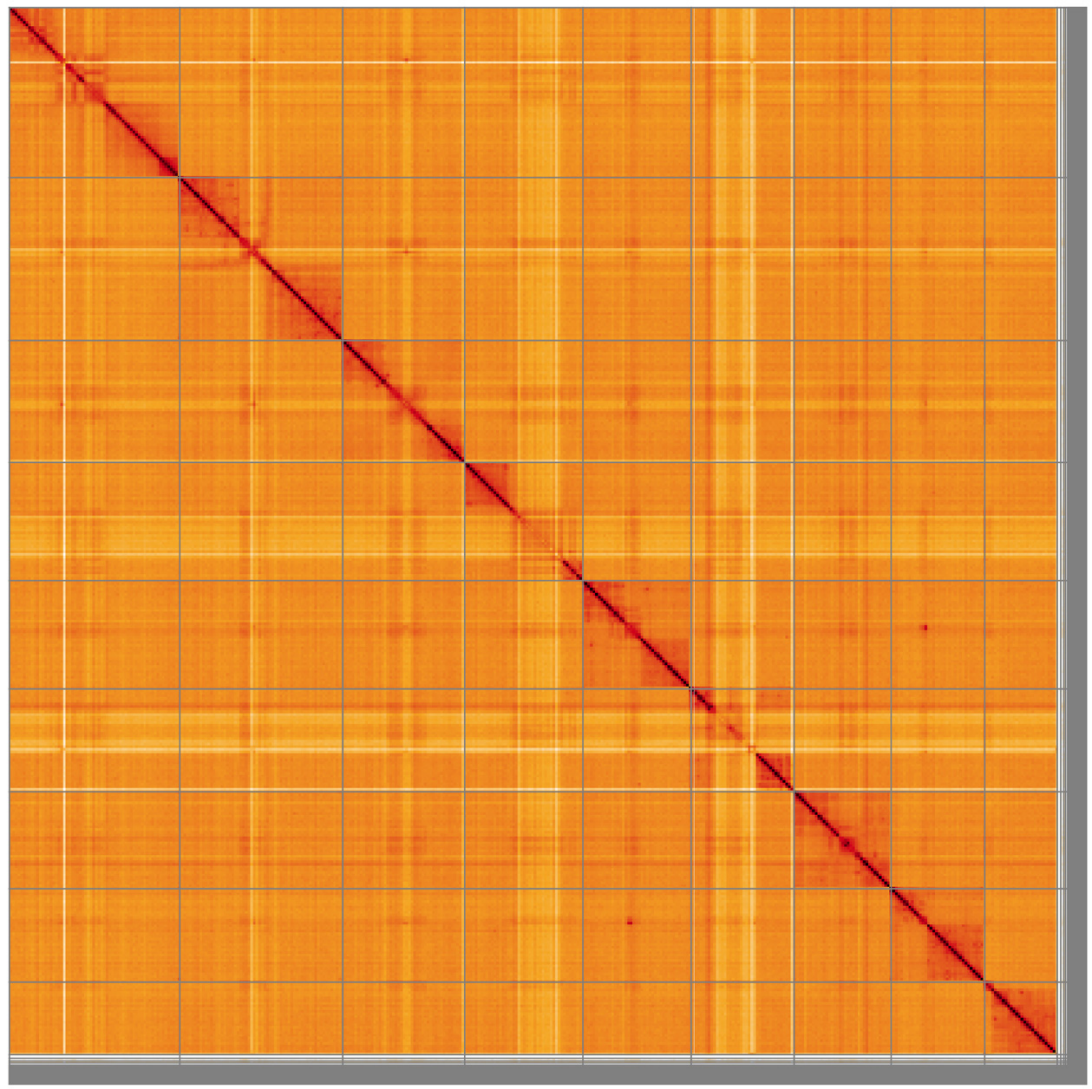
Genome assembly of *Villa cingulata*, idVilCing2.1: Hi-C contact map of the idVilCing2.1 assembly, visualised using HiGlass. Chromosomes are shown in order of size from left to right and top to bottom. An interactive version of this figure may be viewed at https://genome-note-higlass.tol.sanger.ac.uk/l/?d=SXAgkaluToGo3fqjAOJUjw.

The final assembly has a total length of 412.6 Mb in 45 sequence scaffolds with a scaffold N50 of 45.3 Mb ( Table 1). The snailplot in Figure 2 represents a summary of the assembly statistics, while the distribution of assembly scaffolds on GC proportion and coverage is shown in Figure 3. The cumulative assembly plot in Figure 4 shows curves for subsets of scaffolds assigned to different phyla. Most (99.29%) of the assembly sequence was assigned to 10 chromosomal-level scaffolds, representing 8 autosomes and the X and Y sex chromosome. Chromosome-scale scaffolds confirmed by the Hi-C data are named in order of size ( Figure 5, Table 2). The Y chromosome is not scaffolded due to lack of Hi-C signal, as the Hi-C data appears to be from a female sample whereas the PacBio data used for *de novo* assembly is from a male. ( Figure 2– Figure 5; Table 2). While not fully phased, the assembly deposited is of one haplotype. Contigs corresponding to the second haplotype have also been deposited. The mitochondrial genome was also assembled and can be found as a contig within the multifasta file of the genome submission.

**Table 1.** Genome data for *Villa cingulata*, idVilCing2.1.

Project accession data		
Assembly identifier	idVilCing2.1	
Species	*Villa cingulata*	
Specimen	idVilCing2	
NCBI taxonomy ID	2753613	
BioProject	PRJEB57894	
BioSample ID	SAMEA14448141	
Isolate information	idVilCing2, male: thorax (DNA sequencing) idVilCing1, female: head and thorax (Hi-C scaffolding)	
Assembly metrics *		*Benchmark*
Consensus quality (QV)	66.9	*≥ 50*
*k*-mer completeness	100%	*≥ 95%*
BUSCO **	C:94.5%[S:93.5%,D:1.0%], F:1.1%,M:4.4%,n:3,285	*C ≥ 95%*
Percentage of assembly mapped to chromosomes	99.29%	*≥ 95%*
Sex chromosomes	X and Y chromosomes	*localised homologous pairs*
Organelles	Mitochondrial genome assembled	*complete single alleles*
Raw data accessions		
PacificBiosciences SEQUEL II	ERR10662019	
Hi-C Illumina	ERR10614877	
Genome assembly		
Assembly accession	GCA_951394055.1	
*Accession of alternate haplotype*	GCA_951394075.1	
Span (Mb)	412.6	
Number of contigs	152	
Contig N50 length (Mb)	5.8	
Number of scaffolds	45	
Scaffold N50 length (Mb)	45.3	
Longest scaffold (Mb)	65.2	

* Assembly metric benchmarks are adapted from column VGP-2020 of “Table 1: Proposed standards and metrics for defining genome assembly quality” from ( Rhie *et al.*, 2021). ** BUSCO scores based on the diptera_odb10 BUSCO set using v5.3.2. C = complete [S = single copy, D = duplicated], F = fragmented, M = missing, n = number of orthologues in comparison. A full set of BUSCO scores is available at https://blobtoolkit.genomehubs.org/view/Villa%20cingulata/dataset/CATOAS01/busco.

**Table 2.** Chromosomal pseudomolecules in the genome assembly of *Villa cingulata*, idVilCing2.

INSDC accession	Chromosome	Length (Mb)	GC%
OX596018.1	1	62.38	25.5
OX596019.1	2	46.73	27.0
OX596020.1	3	45.25	28.0
OX596021.1	4	41.52	26.0
OX596022.1	5	39.4	29.0
OX596023.1	6	37.12	27.0
OX596024.1	7	35.76	25.5
OX596025.1	8	27.82	26.0
OX596017.1	X	65.15	26.0
OX596026.1	Y	1.37	28.5
OX596027.1	MT	0.02	19.5

The estimated Quality Value (QV) of the final assembly is 66.9 with *k*-mer completeness of 100%, and the assembly has a BUSCO v5.3.2 completeness of 94.5% (single = 93.5%, duplicated = 1.0%), using the diptera_odb10 reference set ( *n* = 3,285).

Metadata for specimens, spectral estimates, sequencing runs, contaminants and pre-curation assembly statistics can be found at https://links.tol.sanger.ac.uk/species/2753613.

## Methods

### Sample acquisition and nucleic acid extraction

A male *Villa cingulata* (specimen ID NHMUK014543879, individual idVilCing2) was netted in Holtspur Bottom Nature Reserve, England, UK (latitude 51.61, longitude –0.67) on 2021-02-13. The specimen was collected and identified by Sue Taylor (Dipterists Forum) and preserved by freezing at –80°C. The specimen used for Hi-C sequencing (specimen ID Ox000478, ToLID idVilCing1) was netted in Wytham Woods, Oxfordshire (biological vice-county Berkshire), UK (latitude 51.77, longitude –1.34) on 2020-06-15. The specimen was collected and identified by Liam Crowley (University of Oxford) and snap-frozen on dry ice.

DNA was extracted at the Tree of Life laboratory, Wellcome Sanger Institute (WSI). The idVilCing2 sample was weighed and dissected on dry ice with tissue set aside for Hi-C sequencing. Thorax tissue was disrupted using a Nippi Powermasher fitted with a BioMasher pestle. High molecular weight (HMW) DNA was extracted using the Qiagen MagAttract HMW DNA extraction kit. HMW DNA was sheared into an average fragment size of 12–20 kb in a Megaruptor 3 system with speed setting 30. Sheared DNA was purified by solid-phase reversible immobilisation using AMPure PB beads with a 1.8X ratio of beads to sample to remove the shorter fragments and concentrate the DNA sample. The concentration of the sheared and purified DNA was assessed using a Nanodrop spectrophotometer and Qubit Fluorometer and Qubit dsDNA High Sensitivity Assay kit. Fragment size distribution was evaluated by running the sample on the FemtoPulse system.

### Sequencing

Pacific Biosciences HiFi circular consensus DNA sequencing libraries were constructed according to the manufacturers’ instructions. DNA sequencing was performed by the Scientific Operations core at the WSI on a Pacific Biosciences SEQUEL II (HiFi) instrument. Hi-C data were also generated from head and thorax tissue of idVilCing1 using the Arima2 kit and sequenced on the HiSeq X Ten instrument.

### Genome assembly, curation and evaluation

Assembly was carried out with Hifiasm ( Cheng *et al.*, 2021) and haplotypic duplication was identified and removed with purge_dups ( Guan *et al.*, 2020). The assembly was then scaffolded with Hi-C data ( Rao *et al.*, 2014) using YaHS ( Zhou *et al.*, 2023). The assembly was checked for contamination and corrected as described previously ( Howe *et al.*, 2021). Manual curation was performed using HiGlass ( Kerpedjiev *et al.*, 2018) and Pretext ( Harry, 2022). The mitochondrial genome was assembled using MitoHiFi ( Uliano-Silva *et al.*, 2023), which runs MitoFinder ( Allio *et al.*, 2020) or MITOS ( Bernt *et al.*, 2013) and uses these annotations to select the final mitochondrial contig and to ensure the general quality of the sequence.

A Hi-C map for the final assembly was produced using bwa-mem2 ( Vasimuddin *et al.*, 2019) in the Cooler file format ( Abdennur & Mirny, 2020). To assess the assembly metrics, the *k*-mer completeness and QV consensus quality values were calculated in Merqury ( Rhie *et al.*, 2020). This work was done using Nextflow ( Di Tommaso *et al.*, 2017) DSL2 pipelines “sanger-tol/readmapping” ( Surana *et al.*, 2023a) and “sanger-tol/genomenote” ( Surana *et al.*, 2023b). The genome was analysed within the BlobToolKit environment ( Challis *et al.*, 2020) and BUSCO scores ( Manni *et al.*, 2021; Simão *et al.*, 2015) were calculated.

Table 3 contains a list of relevant software tool versions and sources.

**Table 3.** Software tools: versions and sources.

Software tool	Version	Source
BlobToolKit	4.1.7	https://github.com/blobtoolkit/blobtoolkit
BUSCO	5.3.2	https://gitlab.com/ezlab/busco
Hifiasm	0.16.1	https://github.com/chhylp123/hifiasm
HiGlass	1.11.6	https://github.com/higlass/higlass
Merqury	MerquryFK	https://github.com/thegenemyers/MERQURY.FK
MitoHiFi	2	https://github.com/marcelauliano/MitoHiFi
PretextView	0.2	https://github.com/wtsi-hpag/PretextView
purge_dups	1.2.3	https://github.com/dfguan/purge_dups
sanger-tol/genomenote	v1.0	https://github.com/sanger-tol/genomenote
sanger-tol/readmapping	1.1.0	https://github.com/sanger-tol/readmapping/tree/1.1.0
YaHS	1.1a.2	https://github.com/c-zhou/yahs

### Wellcome Sanger Institute – Legal and Governance

The materials that have contributed to this genome note have been supplied by a Darwin Tree of Life Partner. The submission of materials by a Darwin Tree of Life Partner is subject to the **‘Darwin Tree of Life Project Sampling Code of Practice’**, which can be found in full on the Darwin Tree of Life website here. By agreeing with and signing up to the Sampling Code of Practice, the Darwin Tree of Life Partner agrees they will meet the legal and ethical requirements and standards set out within this document in respect of all samples acquired for, and supplied to, the Darwin Tree of Life Project. 

Further, the Wellcome Sanger Institute employs a process whereby due diligence is carried out proportionate to the nature of the materials themselves, and the circumstances under which they have been/are to be collected and provided for use. The purpose of this is to address and mitigate any potential legal and/or ethical implications of receipt and use of the materials as part of the research project, and to ensure that in doing so we align with best practice wherever possible. The overarching areas of consideration are:

• Ethical review of provenance and sourcing of the material

• Legality of collection, transfer and use (national and international) 

Each transfer of samples is further undertaken according to a Research Collaboration Agreement or Material Transfer Agreement entered into by the Darwin Tree of Life Partner, Genome Research Limited (operating as the Wellcome Sanger Institute), and in some circumstances other Darwin Tree of Life collaborators.

## Data Availability

European Nucleotide Archive: *Villa cingulata*. Accession number PRJEB57894; https://identifiers.org/ena.embl/PRJEB57894. ( Wellcome Sanger Institute, 2023) The genome sequence is released openly for reuse. The *Villa cingulata* genome sequencing initiative is part of the Darwin Tree of Life (DToL) project. All raw sequence data and the assembly have been deposited in INSDC databases. The genome will be annotated using available RNA-Seq data and presented through the Ensembl pipeline at the European Bioinformatics Institute. Raw data and assembly accession identifiers are reported in Table 1.

## Author information

Members of the Natural History Museum Genome Acquisition Lab are listed here: https://doi.org/10.5281/zenodo.4790042.

Members of the University of Oxford and Wytham Woods Genome Acquisition Lab are listed here: https://doi.org/10.5281/zenodo.4789928.

Members of the Darwin Tree of Life Barcoding collective are listed here: https://doi.org/10.5281/zenodo.4893703.

Members of the Wellcome Sanger Institute Tree of Life programme are listed here: https://doi.org/10.5281/zenodo.4783585.

Members of Wellcome Sanger Institute Scientific Operations: DNA Pipelines collective are listed here: https://doi.org/10.5281/zenodo.4790455.

Members of the Tree of Life Core Informatics collective are listed here: https://doi.org/10.5281/zenodo.5013541.

Members of the Darwin Tree of Life Consortium are listed here: https://doi.org/10.5281/zenodo.4783558.
