# Supplementary material for: The genome sequence of the Downland Villa bee-fly, Villa cingulata (Meigen, 1804)
Source: Wellcome Open Res. 2023 Nov 14;8:526. [Version 1] doi: 10.12688/wellcomeopenres.20123.1 (PMC11301140; doi:10.12688/wellcomeopenres.20123.1)
Supplement: Peer review history

# Reviewer report: Reviewer response for version 1

**DOI:** 10.21956/wellcomeopenres.22285.r89384

**Published:** 2024-08-06

**Authors:** Luciano Cosme

**Affiliations:** 1 Yale University, New Haven, CT, USA

This manuscript presents the genome assembly of the *Villa cingulata*, commonly known as the Downland Villa bee-fly. The genome sequence is 412.6 megabases, scaffolded into 10 chromosomal pseudomolecules, including the X and Y sex chromosomes. The mitochondrial genome has also been assembled, with a length of 22.43 kilobases. 

**Replication Concerns:** The manuscript lacks sufficient detail on how to replicate the findings using the raw data. Although the authors mention the sequencing methods and tools used, they do not provide a detailed workflow or pipeline for processing the raw data to achieve the same genome assembly results.

**Recommendation for Authors:** Include a supplementary section or a dedicated repository that outlines a step-by-step protocol for processing the raw data, including all software parameters, commands, and scripts used. This will enable other researchers to replicate the analysis and verify the results. A detailed methods section will enhance transparency and reproducibility, which are crucial for scientific research.

 Does the project cover genome annotation?

Are sufficient details of methods and materials provided to allow replication by others?

No

Is the rationale for creating the dataset(s) clearly described?

Yes

Are the datasets clearly presented in a useable and accessible format?

Partly

Are the protocols appropriate and is the work technically sound?

Yes

Reviewer Expertise:

Genomics and Population genetics

I confirm that I have read this submission and believe that I have an appropriate level of expertise to confirm that it is of an acceptable scientific standard, however I have significant reservations, as outlined above.

# Reviewer report: Reviewer response for version 1

**DOI:** 10.21956/wellcomeopenres.22285.r89387

**Published:** 2024-08-05

**Authors:** Christophe Klopp

**Affiliations:** 1 INRAE, Castanet Tolosan, France

The article presents read sets and a principal genome assembly for Villa cingulata species. The assembly contigs were built with hifiasm which is the most commonly used assembler for PacBio HiFi reads. The contigs were scaffolded using Hi-C reads with YaHS. The assembly is of high quality. The authors should check the presence of telomers at both ends of their chromosomes.

Are sufficient details of methods and materials provided to allow replication by others?

Yes

Is the rationale for creating the dataset(s) clearly described?

Yes

Are the datasets clearly presented in a useable and accessible format?

Yes

Are the protocols appropriate and is the work technically sound?

Yes

Reviewer Expertise:

Transcriptome and genome assembly and annotation

I confirm that I have read this submission and believe that I have an appropriate level of expertise to confirm that it is of an acceptable scientific standard.

# Reviewer report: Reviewer response for version 1

**DOI:** 10.21956/wellcomeopenres.22285.r89382

**Published:** 2024-08-01

**Authors:** Yiyuan Li

**Affiliations:** 1 Ningbo University, Ningbo, Zhejiang, China

In this paper, the authors assembled the genome of a male Downland Villa bee-fly, *Villa cingulata*. They assembled the 10 chromosomes including the X and Y chromosomes. The genome has been manually curated with high quality. The authors have also included the picture of the specimen they sequenced with detailed information on the collecting time and location. The manuscript is well written with very useful details of the species and its genome. I only have two minor comments:

 1. “the three UK Villa species are very similar in appearance” Would it be possible to distinguish the three species using the COI gene? If so, as the authors already assembled and annotated its mitochondrial genome, it would be helpful to search for the COI gene on the Barcode of Life Database (https://www.boldsystems.org/) or NCBI NT database (https://blast.ncbi.nlm.nih.gov/Blast.cgi) to confirm the taxonomy of the sequenced specimen.

 2. Hifiasm has a few parameters during assembly. It would be helpful to also include the parameters they use for assembling. For example, did the authors use --primary and HiC options (--h1 and --h2) for their assembly?

Are sufficient details of methods and materials provided to allow replication by others?

Yes

Is the rationale for creating the dataset(s) clearly described?

Yes

Are the datasets clearly presented in a useable and accessible format?

Yes

Are the protocols appropriate and is the work technically sound?

Yes

Reviewer Expertise:

Comparative genomics

I confirm that I have read this submission and believe that I have an appropriate level of expertise to confirm that it is of an acceptable scientific standard.
